# Frequent overexpression of AMAP1, an Arf6 effector in cell invasion, is characteristic of the MMTV-PyMT rather than the MMTV-Neu human breast cancer model

**DOI:** 10.1186/s12964-017-0212-z

**Published:** 2018-01-05

**Authors:** Yutaro Otsuka, Tsukasa Oikawa, Hinako Yoshino, Shigeru Hashimoto, Haruka Handa, Hiroki Yamamoto, Ari Hashimoto, Hisataka Sabe

**Affiliations:** Department of Molecular Biology, Graduate School of Medicine, Hokkaido University, North 15, West 7, Kita-ku, Sapporo, Hokkaido 060-8638 Japan

**Keywords:** MMTV-PyMT mice, MMTV-Neu mice, Luminal B-type of human breast cancer, The Arf6-based pathway, AMAP1

## Abstract

**Background:**

The small GTPase Arf6 and its downstream effector AMAP1 (also called ASAP1/DDEF1) constitute a signaling pathway promoting cell invasion, in which AMAP1 interacts with several different proteins, including PRKD2, EPB41L5, paxillin, and cortactin. Components of this pathway are often overexpressed in human breast cancer cells, to be correlated with poor prognosis of the patients, whereas overexpression of the Arf6 pathway did not correlate with the four main molecular classes of human breast tumors. In this pathway, receptor tyrosine kinases, including EGFR and Her2, activate Arf6 via GEP100. MMTV-PyMT mice and MMTV-Neu mice are well-established models of human breast cancer, and exhibit the early dissemination and the lung metastasis, by utilizing protein tyrosine phosphorylation for oncogenesis. PyMT-tumors and Neu-tumors are known to have overlapping gene expression profiles, which primarily correspond to the luminal B-type of human mammary tumors, although they differ in the time necessary for tumor onset and metastasis. Given the common usage of protein tyrosine phosphorylation, as well as the frequent use of these animal models for studying breast cancer at the molecular level, we here investigated whether mammary tumors in these mouse models utilize the Arf6-based pathway for invasion.

**Methods:**

Expression levels of Arf6, AMAP1, and GEP100 were analyzed in PyMT-tumors and Neu-tumors by western blotting. Expression of Arf6 and AMAP1 was also analyzed by immunohistochemistry. The involvement of AMAP1 in invasion, and the possible correlation of its high expression levels with cancer mesenchymal properties were also investigated.

**Results:**

We found that PyMT-tumors, but not Neu-tumors, frequently overexpress AMAP1 and use it for invasion, whereas both types of tumors expressed Arf6 and GEP100 at different levels. High levels of the AMAP1 expression among PyMT-tumor cells were frequently correlated with loss of the epithelial marker CK8 and also with expression of the mesenchymal marker vimentin both at the primary sites and at sites of the lung metastases.

**Conclusions:**

PyMT-tumors appear to frequently utilize the Arf6-based invasive machinery, whereas Neu-tumors do not. Our results suggest that MMTV-PyMT mice, rather than MMTV-Neu mice, are useful to study the Arf6-based mammary tumor malignancies, as a representative model of human breast cancer.

**Electronic supplementary material:**

The online version of this article (10.1186/s12964-017-0212-z) contains supplementary material, which is available to authorized users.

## Background

Breast cancer is the second and the fifth leading cause of tumor-associated deaths in Western societies and in Japan, respectively [1, 2]. As recurrence is the major cause of death from breast cancer, basic mechanisms that underlie recurrence, i.e., tumor early dissemination, invasion, and metastasis, as well as resistance to therapies have been extensively studied using genetically manipulated animal models. Such animal models include mice expressing polyomavirus middle T-antigen (PyMT), which activates Src-family tyrosine kinases [3, 4], or expressing Neu/ErbB2 receptor tyrosine kinase under the control of the mouse mammary tumor virus (MMTV) promoter (i.e., MMTV-PyMT mice and MMTV-Neu mice, respectively). These two types of mice share similar gene expression profiles, which correspond to the luminal B-type of human breast cancer [4, 5]. They generate multifocal adenocarcinomas in the mammary glands and develop lung metastases, but their onset of tumor formation and metastasis differ significantly; MMTV-PyMT mice start to develop primary tumors at 9 weeks of age and develop lung metastasis after 14 weeks of age, whereas MMTV-Neu mice start to develop primary tumors at 18 weeks of age and develop lung metastasis after 27–33 weeks of age [6, 7]. Consequently, PyMT-tumor cells demonstrate much higher invasiveness than Neu-tumor cells in vitro.

Arf6 is a small GTPase that primarily mediates the recycling of membrane components at the cell periphery [8]. Arf6 and its downstream effector AMAP1 are often overexpressed in breast cancer cells, and promote invasion, metastasis, and drug resistance [9–20]. The Arf6 pathway appears to drive the epithelial-mesenchymal transition (EMT) of cancer cells; this pathway downregulates E-cadherin and upregulates integrin recycling and focal adhesion dynamics, when AMAP1 binds to PRKD2 and EPB41L5 [15, 17, 19, 20]. Arf6 can be activated by receptor tyrosine kinases (RTKs), including epidermal growth factor receptor (EGFR), by use of the guanine nucleotide-exchanging factor, GEP100 [16]. The Arf6-based pathway is overexpressed in a large population of human breast cancer to be tightly correlated with poor prognosis and poor overall survival of the patients [13, 16, 19], but this overexpression seems to occur rather randomly among the four major subtypes of human breast cancer, and is not statistically correlated with *TP53* mutations [19].

The luminal B-type of human breast cancers expresses high levels of estrogen receptor (ER) and/or progesterone receptor, and is highly proliferative, whereas *TP53* and *RAS* are known to remain intact in most cases [21, 22]. This type of breast cancer can be effectively treated by hormone therapy. Nevertheless, metastatic recurrence occurs frequently in this cancer even long after curative resection of the primary tumor (20%–30% in 5 years) [23–25].

The Arf6-based pathway appears to account for the poor overall survival of approximately 50% of breast cancer patients who die within several years after their initial diagnosis [19, 20]. We here demonstrate that MMTV-PyMT mice, rather than MMTV-Neu mice, are a useful animal model to analyze the Arf6-based pathway.

## Methods

### Animals

MMTV-PyMT (FVB/N-Tg(MMTV-PyVT)634Mul/J, stock no. 002374) mice and MMTV-Neu (FVB/N-Tg(MMTVneu)202Mul/J, stock no. 002376) mice were obtained from the Jackson Laboratory. All animal experiments were performed under a protocol approved by the Animal Care and Use Committee of Hokkaido University (permission no. 14–0056). All mice were reared in self-ventilated cage, provided food and water ad libitum, and maintained with a 12:12-h light dark cycle. The health and behavior of the mice were monitored daily. All efforts were made to minimize suffering.

### Primary cell culture

Resected tumors were washed with phosphate-buffered saline (PBS) and Hanks’ balanced salt solution with antibiotics (100 U/mL penicillin, Meiji Seika; 50 μg/mL streptomycin, Meiji Seika; and 50 μg/mL gentamicin, Sigma-Aldrich), and then minced in Hanks’ balanced salt solution supplemented with 0.01% collagenase (Sigma-Aldrich). After 2 h of incubation at 37 °C, cells were filtrated using a 100-μm cell strainer (Corning). Blood cells and fibroblasts were removed by centrifugation (80 *g*, 5 min, 3 times). Cells were cultured in Dulbecco’s Modified Eagle Medium supplemented with 10% fetal calf serum at 37 °C, 5% CO_2_.

### Reagents

All other chemicals were purchased from Sigma-Aldrich or Wako Pure Chemical Industries, unless otherwise stated.

### Lentivirus and RNA interference

Lentiviruses expressing short hairpin RNA (shRNA) targeting the 3′-untranslated region of *Arf6* mRNA (5′- CCGGAAGGAGAGAAATCCAAA-3′) or the coding regions of *Amap1* mRNA (5′-GACCTGCTGCAGAACCTTATA-3′: Amap1 #1, 5′-AGATGTGTGAATATCTCATTA-3′: Amap1 #2, 5′-CCAGGGACTTACTTGCATTAA-3′: Amap1 #3) were produced by transfection of 293FT cells (Invitrogen) with the shRNA constructs on the pLKO.1-puro vector, the envelope plasmid pMD2.G (Addgene #12259), and the packaging plasmid psPAX2 (Addgene #12260), using Lipofectamine LTX (Invitrogen) according to the manufacturer’s instructions. shRNA-mediated gene silencing was performed by culturing PyMT or Neu cells with the lentivirus solution for more than 24 h. Cells with successful infection were selected by culturing them in the presence of 1 μg/mL of puromycin for more than 48 h.

### Immunoblotting

The mouse monoclonal antibodies against Arf6, vimentin, β-actin, and γ-tubulin and the rabbit polyclonal antibody against Cytokeratin 8 (CK8) were purchased from commercial sources (Arf6, Santa Cruz Biotechnology, Inc.; vimentin, Cell Signaling; β-actin, EMD Millipore; γ-tubulin, Sigma-Aldrich; and CK8, Abcam). Rabbit polyclonal antibodies against AMAP1 and GEP100 were established as described previously [13, 16]. Peroxidase-conjugated donkey antibodies against mouse or rabbit IgGs were purchased from Jackson ImmunoResearch Laboratories, Inc. Immunoblotting analysis was performed as described previously [16] using ECL western detection reagents (GE Healthcare). For quantitative western blotting, an infrared fluorescence imaging system on Odyssey imager was used (normalized to γ-tubulin).

### Reverse transcription (RT)-PCR analysis

RT-PCR was performed as described previously [16]. Briefly, tumors were lysed with Trizol reagent (Thermo Fisher Scientific). Total RNA was purified using Direct-zol RNA Miniprep kit (Zymo research), and aliquots (0.5 to 1 μg) of the RNA were subjected to RT with SuperScript II reverse transcriptase (Thermo Fisher Scientific). PCR was performed with GoTaq Green (Promega). The primers used were as follows: 5’-ATGAGATCTTCAGCCTCCCGGCTCTCCAGTTTT-3′ as an *Amap1* forward; 5’-AGAAAACTTGACAAAAGCGGTGCCAAGGTCAGG-3′ as an *Amap1* reverse; 5’-TTCCGTGTTCCTACCCCCAATGTG-3′ as a *Gapdh* forward; 5’-ATGCCTGCTTCACCACCTTCTTG-3′ as a *Gapdh* reverse.

### Microarray analysis

Differentially expressed genes were examined using the GEO2R tool in the GEO database (GSE3165). GEO2R is an R programming language-based dataset analysis tool.

### Immunohistochemistry

Immunohistochemical staining was performed as described previously [16]. Briefly, specimens were fixed with 3.7% formalin and embedded in paraffin, and then sliced sequentially at a thickness of 3 μm. Samples were deparaffinized with xylene and rehydrated with graded alcohol. After rinsing with tris-buffered saline, samples were processed for antigen retrieval with EDTA buffer (pH 9.0) at 95 °C for 20 min. Samples were incubated with antibodies against Arf6 (1:100) or AMAP1 (1:200), or normal mouse IgG (1:100) at room temperature for 60 min, and the Histofine Simple Stain MAX PO system (Nichirei) was used for visualization. The coloring reaction was performed with 3,3′-diaminobenzidine (Dojin) for 5 min. Hematoxylin was used as a counterstain. A rabbit polyclonal antibody against Arf6 and mouse normal IgG was purchased from commercial sources (Arf6, Aviva; mouse normal IgG, Santa Cruz Biotechnology, Inc.).

### Invasion assay

Invasion assays were performed using BioCoat Matrigel Invasion Chambers (BD Bioscience, 24-well scale, 8 μm pore size), as described previously [16]. Briefly, 10^5^ cells were seeded in the upper chamber with 100 μL of serum-free medium. The lower chamber was filled with 400 μL of complete medium. After 12 h of incubation, cells were fixed with 4% paraformaldehyde for 30 min, and then stained with crystal violet. The numbers of invaded cells were counted in six different areas for each chamber. More than three independent experiments were performed. The Student *t*-test was performed for group comparisons. We confirmed that there was no statistical difference in cell viabilities between groups using CellTiter 96 (Promega).

### Live-cell imaging

Live-cell imaging was performed as described previously [20]. Briefly, 10^4^ cells were seeded on the fibronectin-coated 35 mm glass-bottom dish. After 12 h incubation, live-cell imaging was performed with a confocal laser-scanning microscope using a CFI Plan Apo VC 40 × H oil-immersion objective with an NA of 1.0 under 5% CO_2_ at 37 °C. Cells were exposed to Hoechst 33342 at 1 μg/ml for 30 min before observation to visualize nuclei, and their trajectories were analyzed with the attached software (Model A1R with NIS-Elements, Nikon). Frames were taken every 5 min for 6 h. More than 30 cells from 3 different experiments were analyzed.

### Immunofluorescence

Immunofluorescence staining was performed as described previously [15]. OCT embedded frozen sections (10 μm) were fixed with 4% paraformaldehyde at room temperature for 20 min. After blocking with goat serum in 0.3% Triton X-100 for 30 min, samples were incubated with antibodies against AMAP1 (1:100), CK8 (1:100), and vimentin (1:50) overnight at 4 °C. After rinsing with PBS, samples were incubated with secondary antibodies conjugated with Cy3 or Alexa488 fluorescent dyes for 1 h at room temperature. 4′,6-diamidino-2-phenylindole (DAPI) solution was used for nuclear staining. Samples were mounted with 50% glycerol in PBS, and then subjected to microscopy. The number of tumor cells positive for CK8, vimentin, and/or AMAP1 were counted. More than 100 cells from more than 10 different regions were counted. Alexa488-conjugated goat antibodies against mouse or rabbit IgGs were purchased from Molecular Probes and Cy3-conjugated donkey antibodies against mouse or rabbit IgGs were purchased from Jackson ImmunoResearch Laboratories, Inc.

### Statistical analysis

All statistical analyses were performed using GraphPad Prism software. A *P*-value of less than 0.05 was considered to indicate a statistically significant difference between two groups.

## Results

### Overexpression of AMAP1 in PyMT-tumors

**Fig. 1:**
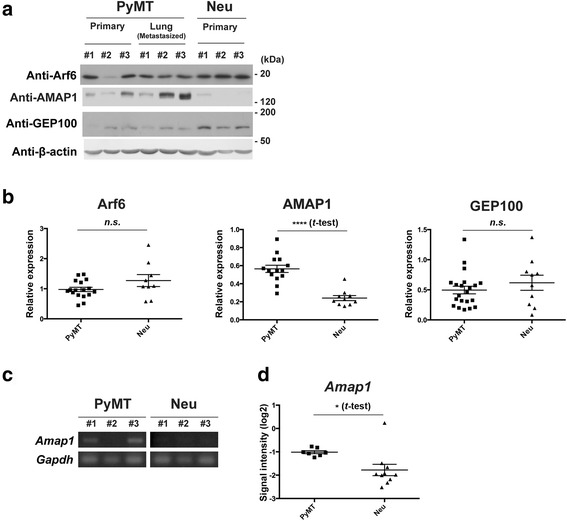
AMAP1 is differentially expressed in tumors from MMTV-PyMT and MMTV-Neu mice. **a** Primary tumors or lung-metastasized tumors from PyMT mice (12–20 weeks old) or Neu mice (40–56 weeks old) were subjected to immunoblot analysis with the indicated antibodies. **b** Quantitative western blots of Arf6, AMAP1, GEP100, and γ-tubulin in primary tumors were performed using an infrared fluorescence imaging system on Odyssey imager (normalized to γ-tubulin). Bars represent means ± SE. ****P < 0.0001 (Student *t*-test); *n.s.*, not significant. **c** Primary tumors from PyMT mice (12–20 weeks old) or Neu mice (40–56 weeks old) were subjected to semi-quantitative RT-PCR analysis with the primers for the indicated genes. **d** The public microarray datasets of PyMT- or Neu-tumor cells (GSE3165) were examined using the GEO2R tool. *Amap1* mRNA levels in these cells are shown. Bars represent means ± SE. *P < 0.05 (Student *t*-test)

We have demonstrated the frequent involvement of the Arf6-based pathway in breast cancer malignancy, as earlier mentioned. However, there is no spontaneous breast cancer model in which this pathway is utilized for malignant development. We first examined the protein expression of Arf6, AMAP1 and GEP100 by western blotting of whole tumor lysates, isolated from MMTV-PyMT mice and MMTV-Neu mice. We found that most PyMT-tumors, either at the primary sites or in the lungs, express these proteins at detectable levels, although the expression levels varied among different preparations (Fig. 1a and b). Neu-tumors also expressed Arf6 and GEP100 at detectable levels, although the expression of AMAP1 was very low or almost undetectable (Fig. 1a and b).

To be consistent with above results, we detected higher levels of *Amap1* mRNA in PyMT-tumor cells than in Neu-tumor cells (Fig. 1c). This result was consistent with the data from the public microarray datasets of these mouse models (Fig. 1d). On the other hand, we have reported previously that the AMAP1 protein levels do not correlate with its mRNA levels among different human breast cancer cells [13]. The *Amap1* mRNA contains a long 5′-untranslated region (UTR) with an internal ribosome entry site (IRES), and also a terminal oligopyrimidine (TOP) sequence [26, 27], suggesting that translational processes of this mRNA might participate in regulating its protein levels. Our above results, however, implied that such translational regulations may not be critical to determine the AMAP1 protein levels in these animal models under our experimental condition.

**Fig. 2:**
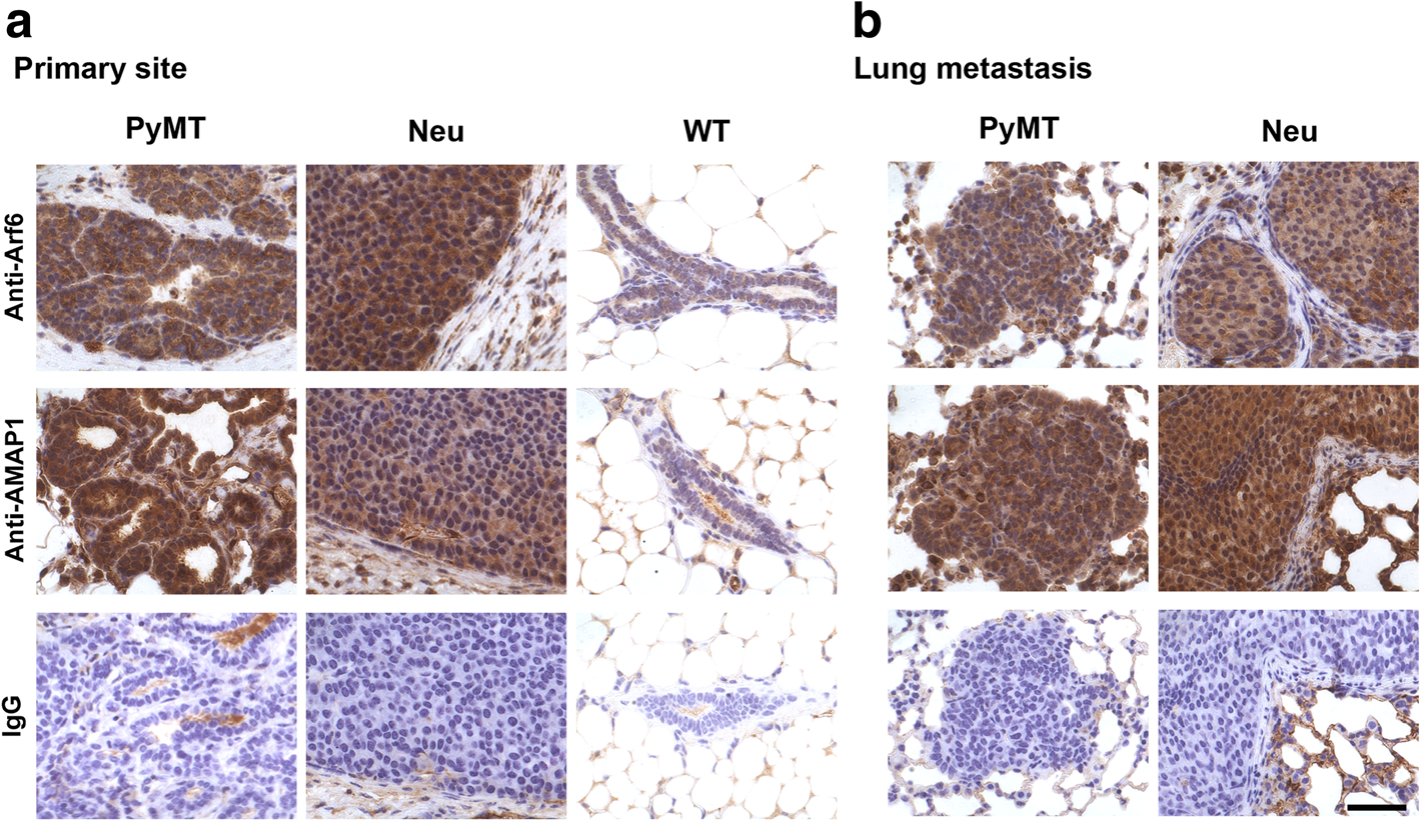
AMAP1 is expressed in cancer cells but not in stromal cells. Primary (**a**) or lung-metastasized (**b**) tumors from the PyMT (12–20 weeks old) or Neu (40–56 weeks old) mice, or mammary glands from wild-type (WT) mice (10 weeks old) were fixed, embedded in paraffin, and stained with an antibody to Arf6 or AMAP1. Normal mouse IgG (IgG) was used as a control for each staining. Nuclei were stained with hematoxylin. Representative images of each tumor are shown. Scale bar, 50 μm

We then performed immunohistochemical analysis and confirmed that Arf6 and AMAP1 are expressed at high levels in cancer cells rather than in stromal cells (Fig. 2a and b). We also confirmed lower levels of AMAP1 expression in Neu-primary tumors than in PyMT-primary tumors (Fig. 2a). At the metastatic sites, ubiquitous expression of AMAP1 was observed in most cases, whereas the expression of Arf6 demonstrated regional variations even within the same tumor lesions both in PyMT- and Neu-tumors (Fig. 2b). On the other hand, moderate expression of these proteins was detected in mammary gland duct cells of wild-type mice (10-weeks old) (Fig. 2a), indicating that their physiological roles are likely associated with tissue remodeling [28]. Therefore, collectively, MMTV-PyMT mice, rather than MMTV-Neu mice, appear to frequently express the Arf6-AMAP1 pathway at high levels in their primary tumors.

MMTV-Neu mice also demonstrate lung metastasis, albeit with lower frequencies than those of MMTV-PyMT mice (see Additional file 1). Interestingly, Neu-tumors at the metastatic sites also express AMAP1, as well as Arf6, at high levels (Fig. 2b).

### AMAP1 is crucial for the invasion of PyMT-tumors

**Fig. 3:**
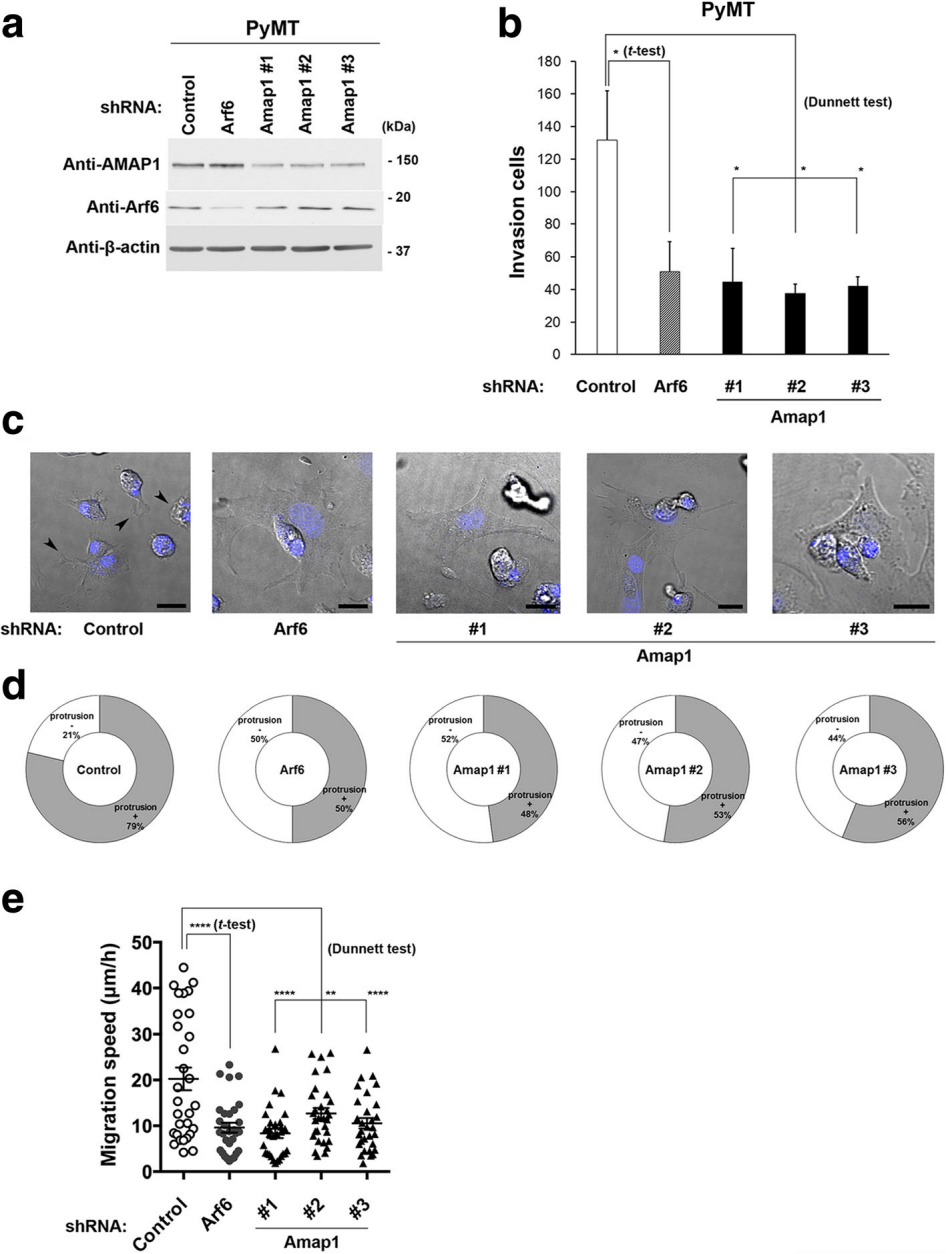
AMAP1 is required for the invasive activity of MMTV-PyMT tumor cells. **a** PyMT tumor cells were transduced with the indicated shRNAs and were subjected to immunoblot analysis with the indicated antibodies. **b** PyMT tumor cells were transduced with the indicated shRNAs and were then transferred to a Matrigel chamber and assayed for invasive activity. Cells that invaded through the Matrigel were stained with crystal violet, and the number of cells in three distinct regions in a single chamber was quantified. Data are means ± SE from more than three independent experiments. *P < 0.05 (Student *t*-test or Dunnett test, as indicated); *n.s.*, not significant. **c** PyMT tumor cells were transduced with the indicated shRNAs and were subjected to live-cell imaging analysis. Nuclei were stained with Hoechst 33342 (blue). Representative differential interference contrast (DIC) images of these cells are shown. Arrowheads indicate membrane protrusions. Scale bar, 20 μm. **d** The cells with (+) or without (−) membrane protrusions were quantified and the percentage of these cells is shown. **e** Velocity of migration obtained by the total track distance of each cell divided by time is indicated. Bars represent means ± SE. **P < 0.01; ****P < 0.0001 (Student *t*-test or Dunnett test, as indicated)

We then analyzed an involvement of AMAP1, as well as Arf6, in invasion of these tumor cells. For this, tumor cells were isolated from the primary tumor lesions and were cultured in vitro, and then transduced with shRNA to silence *Amap1* and *Arf6* (Fig. 3a; also see Additional file 2 and Methods). Cell invasion activities, as measured using the Matrigel invasion chamber, were significantly blocked by all of these shRNAs in PyMT-tumor cells (Fig. 3b), but not in Neu-tumor cells (Additional file 2). Silencing of *Arf6* also blocked the invasion of PyMT-tumor cells (Fig. 3b). Silencing of *Arf6* and *Amap1* also significantly impaired membrane ruffles and lamellipodial protrusions (Fig. 3c and d), and two-dimensional cell migration (Fig. 3e). Thus, AMAP1 appears to be crucial for invasiveness of PyMT-tumor cells, likely via upregulating plasma membrane dynamics to be consistent with a basic role of Arf6 [29].

### Tumor cells with high AMAP1 levels frequently lose the epithelial phenotype

**Fig. 4:**
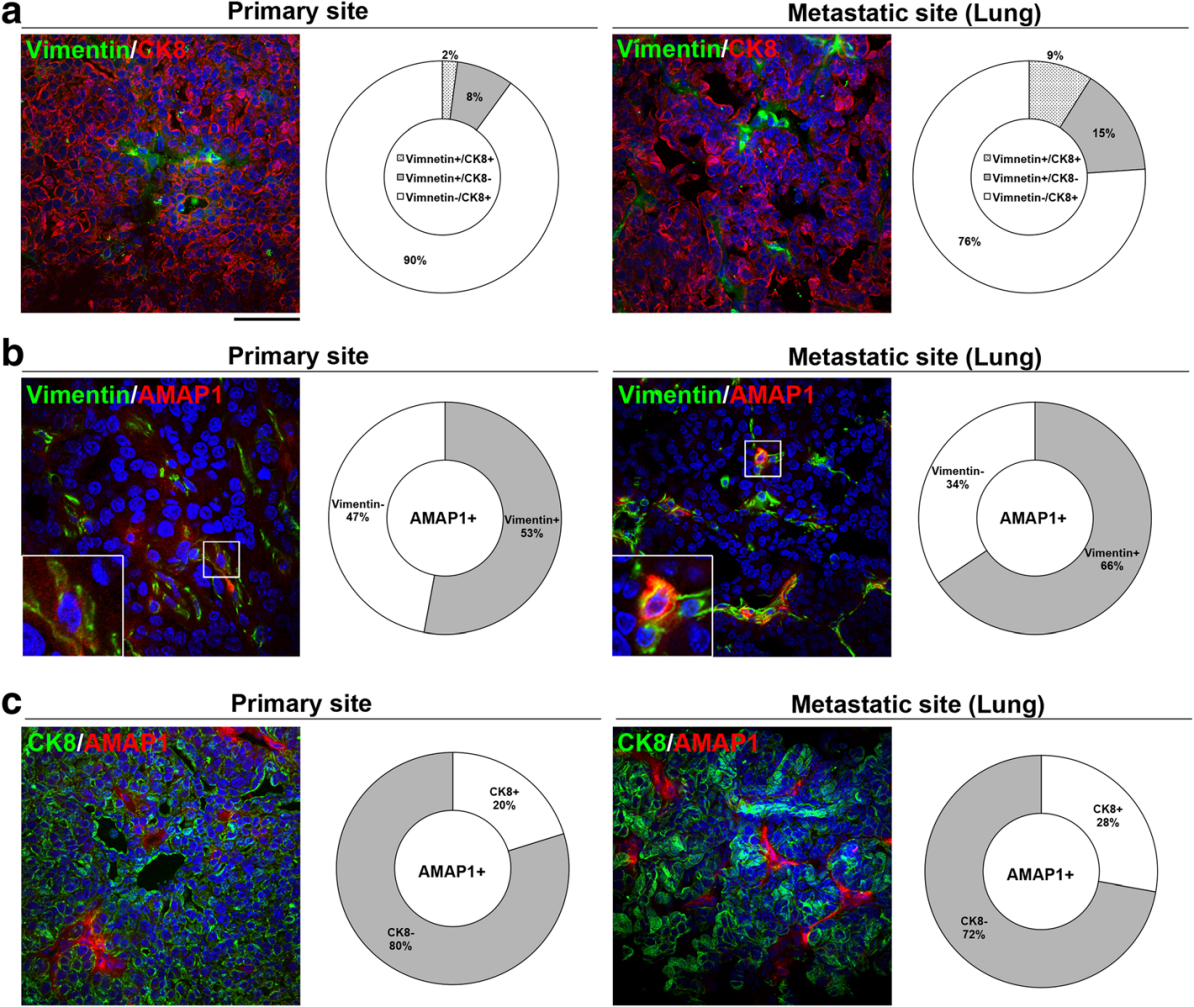
Tumor cells with high AMAP1 expression tend to lose the epithelial phenotype. Primary tumors or tumors from the metastatic site (lung) of PyMT mice (12–20 weeks old) were subjected to immunofluorescence staining with antibodies to CK8 and vimentin (**a**), AMAP1 and vimentin (**b**), or AMAP1 and CK8 (**c**). Nuclei were stained with DAPI (blue). Representative images of each tumor are shown. Scale bar, 50 μm. Quantification of tumor cells positive for CK8, vimentin, and/or AMAP1 are shown on the right of each image. More than 100 cells from more than 10 different regions were counted for each sample

Loss of the epithelial markers and expression of the mesenchymal markers, is a hallmark of cancer EMT. On the other hand, cancer cells, having once undergone EMT, frequently regain their epithelial phenotypes, such as at metastatic sites via mesenchymal-epithelial transition [30, 31]. Expression of the epithelial marker, CK8, was observed in all of PyMT-tumors both at the primary sites and the lungs, although its expression levels varied among tumor cells even within the same tumor mass (Fig. 4a). Moreover, cells expressing the mesenchymal marker, vimentin, and cells expressing CK8 were mutually exclusive in most of these tumor cells (Fig. 4a). Notably, high expression of AMAP1 was observed with the vimentin-positive cells of the primary sites with a frequency of 53%, and this correlation was slightly increased in the lungs (66%) (Fig. 4b). Furthermore, high expression of AMAP1 was well correlated with the absence of CK8 expression both at the primary sites (80%) and in the lungs (72%) (Fig. 4c). These results suggested that the high expression of AMAP1 protein are often coupled with loss of the epithelial phenotype and activation of the cancer mesenchymal program in PyMT-tumors.

## Conclusions

Overexpression of the Arf6-based pathway in breast cancer cells appears to account for the poor overall survival of the majority of patients who die within several years after their diagnosis. In this study, we demonstrated that PyMT-tumors frequently overexpress Arf6 and AMAP1, and utilize them for invasion and metastasis. Thus, MMTV-PyMT mice provide an animal model system to study Arf6-based tumor malignancy. Many studies on metastatic breast cancer, including those on cancer immunity or cancer-specific metabolic rewiring, have been performed using PyMT mice, as seen in a very recent publication [32]. Therefore, the role of the Arf6 pathway in these processes will also be clarified using this mouse model.

Among different human breast cancer cell lines we have examined previously, highly-invasive cells expressed higher amounts of the AMAP1 protein than those of weakly- or non-invasive cells, while all of these cancer cells expressed almost similar levels of the *AMAP1* mRNA [13]. Thus, it was unexpected to us that PyMT-tumors and Neu-tumors exhibit different *Amap1* mRNA levels, to be consistent with the different AMAP1 protein levels. Moreover, high expression of AMAP1 protein often correlated with the loss of CK8, an epithelial marker, in PyMT-tumors. AMAP1 is not a mesenchymal-specific protein. Therefore, molecular bases as to why the high expression of *Amap1* mRNA is specific to PyMT-tumors rather than Neu-tumors, and why high expression of AMAP1 protein often correlate with the loss of CK8 and the activation of EMT program await to be clarified.

The mevalonate pathway (MVP) activity is essential for Arf6 activation, whereas oncogenic-p53 may enhance MVP activity and hence promote Arf6-based tumor malignancy [20]. On the other hand, overexpression of the Arf6-based pathway and its association with poor prognosis can be observed frequently in the presence of intact *TP5*3 [19]. *Trp53* is known to remain intact in PyMT-tumors [33, 34] (we also herein confirmed this). We have shown previously in vitro that combination of conventional anticancer drugs with statins, the MVP inhibitors, is very effective to kill breast cancer cells, if cells overexpress the Arf6-based pathway [19, 20]. Immune activities appear to be critical even to the efficacies of anticancer drugs in vivo [35]. The Arf6 pathway is overexpressed in a large population of the invasive/metastatic breast cancer cells in humans, as earlier mentioned. Therefore, it is now possible to investigate such a potential, but restricted usefulness of statins in cancer therapy by using immune-competent mice, in order to treat the highly-invasive and metastatic breast cancers. The MMTV-PyMT mouse model might also be useful to study the immune-based therapies against the Arf6-overexpressing breast cancer cells. Furthermore, a low cholesterol diet may be effective for cancer treatment [36, 37], although it is also known that a low cholesterol intake upregulates MVP activity [38, 39]. Thus, in the future, the actual effects of these diets on breast cancer malignancy should also be clarified using this mouse model.

## Additional files

Additional file 1: MMTV-PyMT mice develop metastatic tumors at younger ages than MMTV-Neu mice. (A) Kaplan-Meier plots showing tumor-free survival of PyMT and Neu mice. *P* < 0.0001 (log-rank test). (B) The presence of lung metastasis was analyzed in PyMT or Neu mice that have primary tumors greater than 20 mm in diameter. *P* < 0.0001 (Fisher exact test). (TIFF 269 kb)

Additional file 2: The invasive activity of MMTV-Neu tumor cells does not depend on AMAP1. Neu tumor cells were transduced with the indicated shRNAs and were subjected to immunoblot analysis with the indicated antibodies. (B) Neu tumor cells were transduced with the indicated shRNAs and were then transferred to a Matrigel chamber and assayed for invasive activity. Cells that invaded through the Matrigel were stained with crystal violet, and the number of cells in three distinct regions in a single chamber was quantified. Data are means ± SE from more than three independent experiments. *n.s.*, not significant (Student *t*-test or Dunnett test, as indicated). (TIFF 192 kb)

## Abbreviations

CK8: Cytokeratin 8
DAPI: 4′,6-diamidino-2-phenylindole
EGFR: Epidermal growth factor
EMT: Epithelial-mesenchymal transition
ER: Estrogen receptor
IRES: Internal ribosome entry site
MMTV: Mouse mammary tumor virus
MVP: Mevalonate pathway
PBS: Phosphate-buffered saline
PyMT: Polyomavirus middle T antigen
RT: Reverse transcription
RTKs: Receptor tyrosine kinases
shRNA: short hairpin ribonucleic acid
TOP: Terminal oligopyrimidine
UTR: Untranslated region
