# Supplementary figures and images for: Frequent overexpression of AMAP1, an Arf6 effector in cell invasion, is characteristic of the MMTV-PyMT rather than the MMTV-Neu human breast cancer model
Source: Cell Commun Signal. 2018 Jan 5;16:1. doi: 10.1186/s12964-017-0212-z (PMC5795291; doi:10.1186/s12964-017-0212-z)

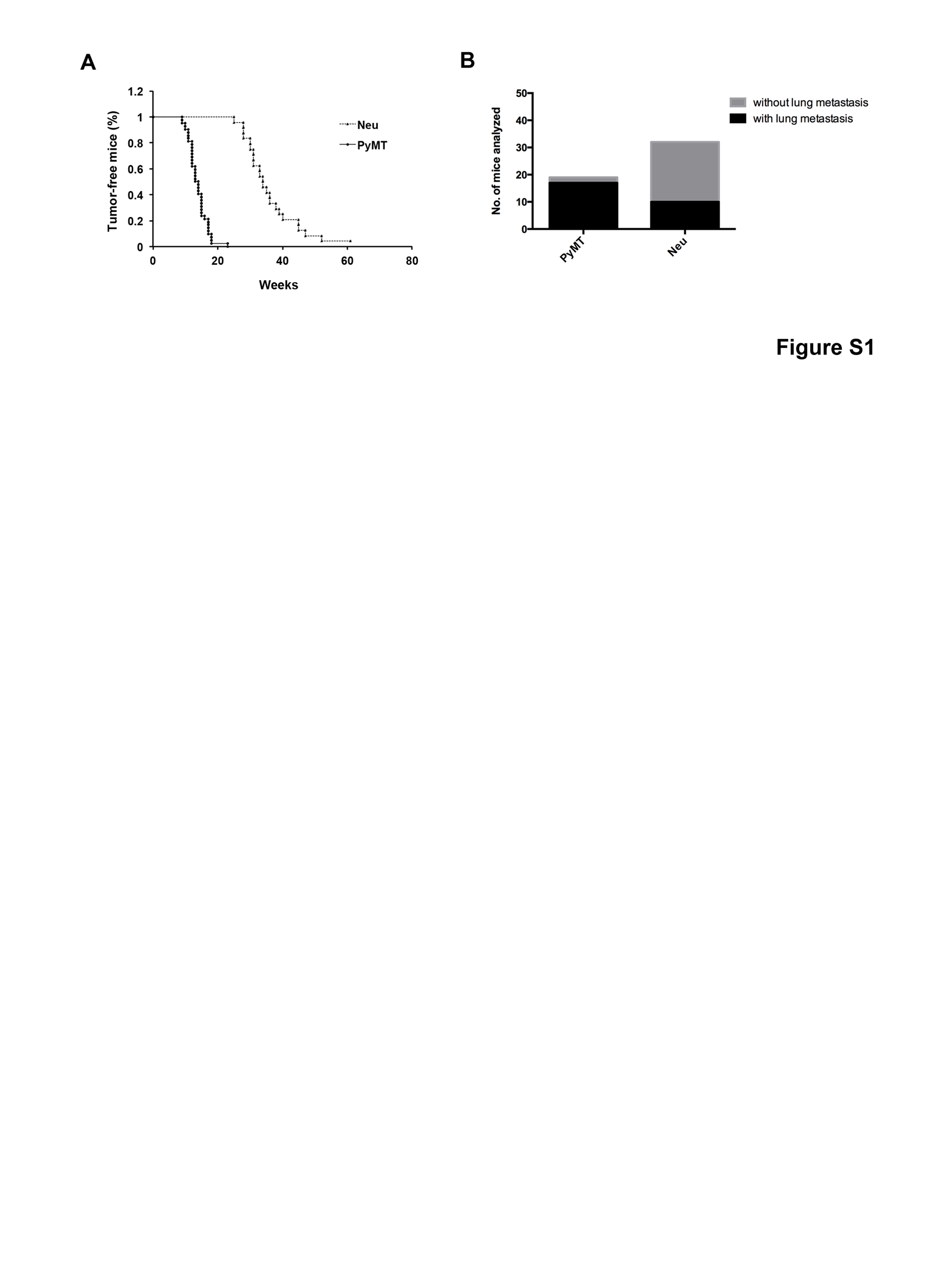

Supplement: Supplementary file 1 — MMTV-PyMT mice develop metastatic tumors at younger ages than MMTV-Neu mice. (A) Kaplan-Meier plots showing tumor-free survival of PyMT and Neu mice. P < 0.0001 (log-rank test). (B) The presence of lung metastasis was analyzed in PyMT or Neu mice that have primary tumors greater than 20 mm in diameter. P < 0.0001 (Fisher exact test). (TIFF 269 kb) [file 12964_2017_212_MOESM1_ESM.tif]

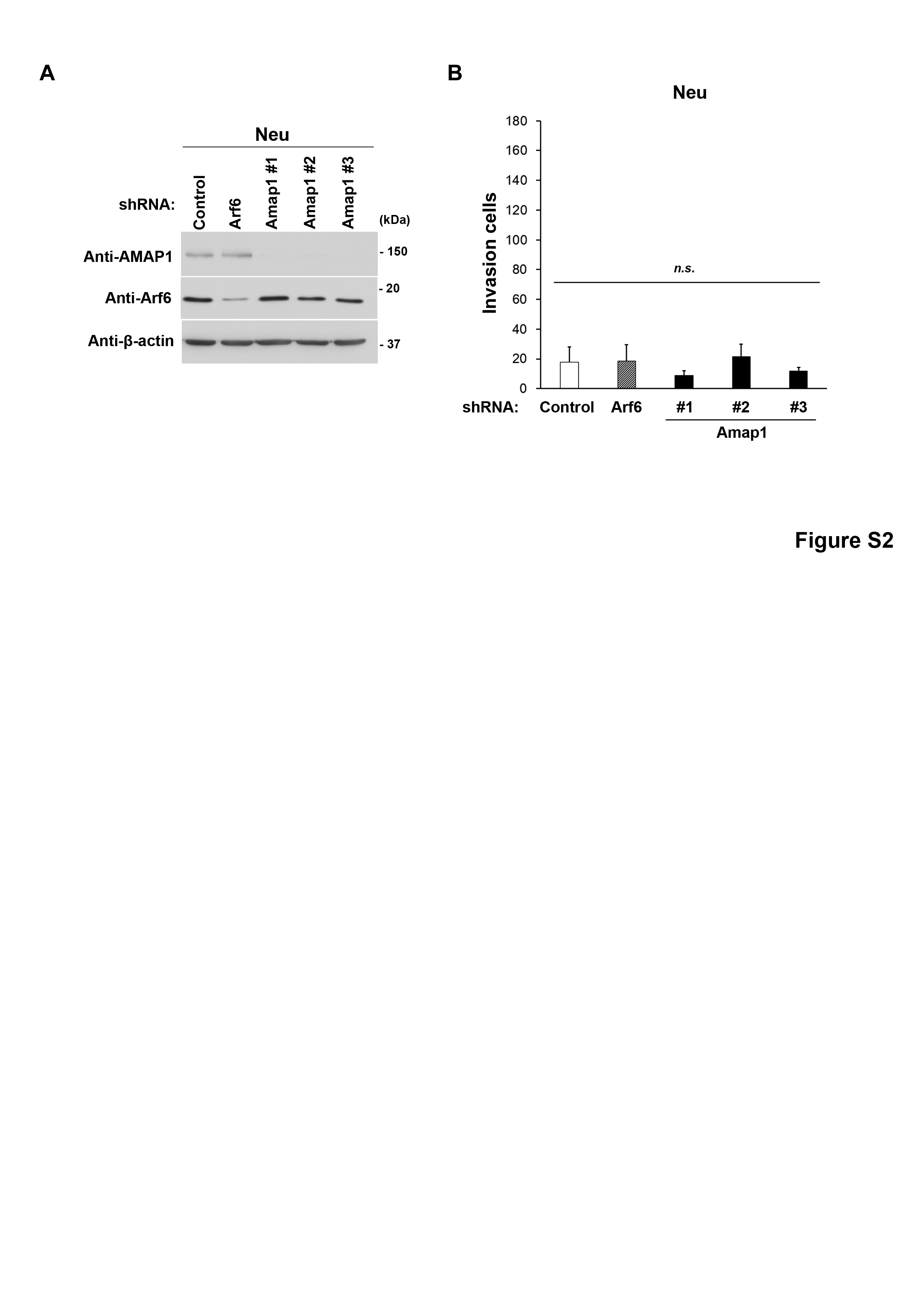

Supplement: Supplementary file 2 — The invasive activity of MMTV-Neu tumor cells does not depend on AMAP1. Neu tumor cells were transduced with the indicated shRNAs and were subjected to immunoblot analysis with the indicated antibodies. (B) Neu tumor cells were transduced with the indicated shRNAs and were then transferred to a Matrigel chamber and assayed for invasive activity. Cells that invaded through the Matrigel were stained with crystal violet, and the number of cells in three distinct regions in a single chamber was quantified. Data are means ± SE from more than three independent experiments. n.s., not significant (Student t-test or Dunnett test, as indicated). (TIFF 192 kb) [file 12964_2017_212_MOESM2_ESM.tif]
